# Heart-lung transplantation: Historical notes

**DOI:** 10.1016/j.jhlto.2026.100643

**Published:** 2026-07-23

**Authors:** John H. Dark

**Affiliations:** Professor of Cardiothoracic Surgery, Translational and Clinical Research Institute, Faculty of Medical Sciences, Newcastle University

**Keywords:** Heart-Lung Transplantation, Lung Transplantation, Domino-Heart Transplant, Organ Preservation, Lung Transplant History

## Background

As we pass the 45th anniversary of the first successful combined heart and lung transplant (on March 8, 1981), it seems useful to consider what we can learn from a historical perspective.

There was the initial experimental development with the early realization that only primates could breathe spontaneously after bilateral denervation of the lung. Then we saw the almost parallel development of isolated lung transplant and the combined heart-lung procedure. The first of each were done within a few years of each other, and the early experience of both procedures was dogged by persistent failure. The emergence of what were to be dominant investigative groups in Stanford and Toronto lead to successful outcomes again within a year or 2 of each other. We then saw the combined heart-lung transplant operation flower into dominance because it was the only successful procedure for those who required replacement of 2 lungs, mainly those with septic lung disease. But with the introduction of the final version of the bilateral lung transplant in 1990, the pendulum swung the other way, and the indications for combined heart-lung transplant are now few, although still debated.

### Early history

These early developments are described in detail by Cooper.^1^ He describes that the story begins at the start of the 20th century, when Carrel transplanted a heart-lung block into the neck of a cat. This was followed by Demikov’s huge range of ingenious experiments, transplanting a heart-lung block into the chest using sequential division and re-suturing of vessels and then the airway to obviate the need for the heart-lung bypass machine. This work was done in dogs, and while some survived several days, it was apparent that the respiratory pattern was abnormal.

The first successful transplants with long-term survival were described in primates by Castaneda et al. This work was expanded by Reitz and colleagues, initially with auto-transplantation and then successful allotransplantation, again in small monkeys. By the end of the series, the Stanford team had access to Cyclosporin A and could achieve long-term survival. This group also perfected the operative approach to implantation, which has remained largely unchanged.^2^

The first clinical heart-lung transplant had been done by Cooley et al in 1968,^3^ followed by single cases done by Lillehei^4^ in1969 and Barnard^5^ in 1971. None of these patients survived for more than a few weeks.

But based on their meticulous set of monkey experiments, the group lead by Reitz performed their first clinical transplant in March 1981. There was good early function and, benefitting from Cyclosporin-A, the patient survived for many years.^6^

### Evolution

In contrast with the rapid, if unsuccessful, adoption of isolated heart and isolated lung transplant in the late 60’s/early, growth of activity was slow. The ISHLT registry, reporting on heart-lung transplants in 2010, records only a handful of these transplants in 1982, 1983, and 1984, rising to perhaps 40, throughout the world in 1985.^7^ After Stanford, Pittsburgh was an active center in the USA, with 33 procedures in a little under 3 years to 1986^8^ The first heart-lung transplant in Europe was from the team of Wellens in Belgium, August 1983,^9^ followed by Yacoub in London in 1984.^10^

### The golden age

**Figure 1:**
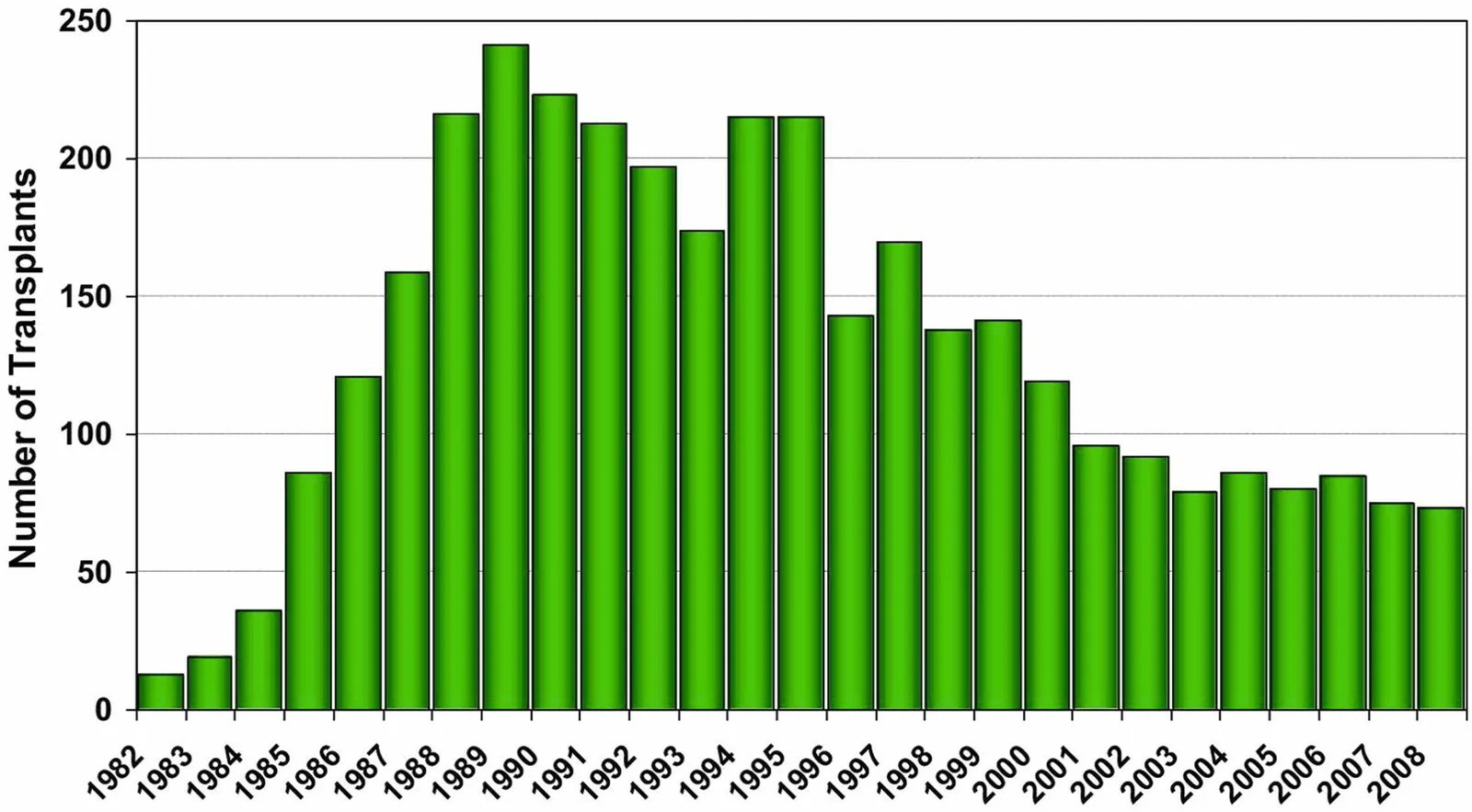
*Number of heart-lung transplants reported by year.* Taken from the ISHLT Annual Registry Report, 2010, J Heart Lung Transplant. 2010 Oct; 29^10^: 1083–1141.

There was, for a while, an absence of any other reliable pulmonary transplant procedure for patients with pulmonary hypertension, or lung sepsis, such as Cystic fibrosis. From well under 50 cases per year in 1985, activity soared to almost 250 cases a year in the ISHLT registry in 1990,^7^ Figure 1 There was then a progressive fall over the next 10 to 12 years, to less than 100 cases from all centers in the world.

Several groups had shown that in the absence of lung sepsis, single lung transplantation could safely be performed for both obstructive lung disease and pulmonary hypertension. In the latter, recovery of right ventricular function, already seen after pulmonary thrombo-endarterectomy, was reliably reported—a response to normalizing pulmonary vascular resistance.

But for patients with sepsis, the approach remained predominantly that of heart and lung transplantation. This was despite the perception that the heart was largely normal in most of these patients. Whilst the en-bloc double lung transplant was reported in 1986^11^ it was dogged by airway ischemia as well as the need for full cardiopulmonary bypass and an extensive dissection. Various European groups contributed with progressively distal bronchial, rather than tracheal anastomoses.^12^ But the big step forward was from the group in St Louis, now lead by Cooper. They moved all the anastomoses to the lung hilum and in particular, put the bronchial anastomosis as close as possible to the lung parenchyma. This step, in combination with the clamshell rather than sternotomy, gave better access to any pleural adhesions (common in septic lung disease) and permitted off-pump, “sequential” lung transplantation.^13^ It is notable that a French group working independently described the same procedure at the same time^14^

By 1993, when the number of heart-lungs in the ISHLT registry had fallen to less than 150, there were already double that number of bilateral lung transplants.^7^ 30 years later, the number of bilateral lung transplants reported to the registry exceeded 3,500 in a year when the total, adult and pediatric, for heart-lungs lay between 50 and 100 per year with little change from decade to decade.

### Indications

As well as the established role of the bilateral lung transplant, this huge difference follows from alterations in access to heart-lung blocks, with allocation systems throughout the world favoring separate cardiac and paired lung transplants. In the current era, many recipients have both a background of congenital heart disease with pulmonary hypertension. Others have pulmonary hypertension in a setting that will not permit isolated lung transplants. The current situation is well captured by a recent review from Stanford, with useful comparisons between what they call the “pioneer era” (up to 2001) and the past 20+ years.^15^

As we conclude these notes on the history of the procedure, there are some features which are specific to the heart-lung transplant.

### The domino-procedure

A constant problem has been the perception that the heart-lung transplant takes an unfair allocation of donor organs: it requires both the heart and the lungs. One option to obviate this problem, particularly when transplanting many patients with predominant respiratory disease, is to offer on the heart lung transplant recipient’s heart to a cardiac transplant patient. This was first performed by Yacoub in 1988^16^ and then initially in the United States at John’s Hopkins University a year later.^17^ There were clearly logistical demands, with 2 major transplants occurring usually in adjacent operating rooms. But the procedure did maximize use of the donor organs. There were some advantages in because the heart from the heart-lung recipient often had some preparation of the right ventricle (related to the underlying respiratory disease) In addition, the heart had often a short ischemic time and had not gone through the stress of brain death in the donor. Perhaps because of this, the long-term results from the Harefield series showed good long-term survival and relatively little graft vasculopathy.^18^ Despite this, the thoracic surgical community were recorded as describing this use of the recipient’s heart as “theft.”^19^ However, as the heart-lung procedure became focused on patients with severe pulmonary vascular disease and cardiac abnormalities, the domino transplant almost disappeared. Thus, the last classical “domino” transplant at Stanford was in 1994, and the last in the US in 1996.^20^

### Donor organ retrieval

A surprising variation in organ preservation has been observed. For the initial Stanford series, the donor and recipient were in adjacent operating rooms. The heart received standard cold cardioplegia and the lungs a flush with the then solution of choice, Euro-Collins. Yacoub’s first heart-lung was done in the same fashion.

Because of concerns about ischemic damage, particularly with the early lung flushing solutions and the need for distant retrieval, the group in Pittsburgh developed an autoperfusing heart-lung block. The lungs were inflated and the heart continued to beat, supplied by a reservoir raised above the right atrium. There was both pulmonary artery and coronary perfusion with oxygenated blood. This ingenious system both allowed a visual assessment of the function of the heart and lungs and permitted ischemic times out to 7 hours. It predated by decades the current enthusiasm for organ perfusion during transport.^21^

Another approach, that of placing the donor on bypass at the donor hospital and cooling to preserve both heart and lungs, was successful in the hands of Yacoub and colleagues.^22^ Again, a technique used in the 1980’s preceded, in concept, by decades some current approaches to the problem of organ ischemia after donation after circulatory death.

## Conclusion

The history of the combined heart-lung transplant allows us to see the evolution of a technique and the demonstration of success in animal models. It moved cautiously into clinical practice but became, for a while, the only successful approach for many patients with a range of pulmonary diseases. Overtaken by parallel developments in the bilateral transplant, it remains a proven approach for a carefully selected subset of patients requiring both heart and lung replacement for failure of both organs. Techniques such as the domino transplant, or the auto-perfusing transport circuit, should not be forgotten as we continue to move forwards in the treatment of patients with end-stage cardiopulmonary disease.

## Conflicts of Interest statement

The authors declare that they have no known competing financial interests or personal relationships that could have appeared to influence the work reported in this paper.
